# Supplementary material for: Unveiling the Interfacial Reconstruction Mechanism Enabling Stable Growth of the Delafossite PdCoO2 on Al2O3 and LaAlO3
Source: ACS Appl Mater Interfaces. 2025 Apr 14;17(16):24620–9. doi: 10.1021/acsami.5c03536 (PMC12022947; doi:10.1021/acsami.5c03536)
Supplement: Supplementary file 1 — am5c03536_si_001.pdf [file am5c03536_si_001.pdf]

Supporting Information:

Unveiling the Interfacial Reconstruction Mechanism Enabling Stable Growth of the Delafossite $PdCoO_2$ on Al_2O_3 and $LaAlO_3$

Anna Scheid[1,*], Tobias Heil[1], Y. Eren Suyolcu[1], Qi Song[2], Niklas Enderlein[3], Arnaud P. Nono Tchiomo[4], Prosper Ngabonziza[4,5], Philipp Hansmann[3], Darrell G. Schlom[2,6,7], and Peter A. van Aken[1]

[1]Max Planck Institute for Solid State Research, Stuttgart, 70569, Germany

[2]Department of Materials Sciences and Engineering, Cornell University, Ithaca, New York 14853, USA

[3]Department of Physics, Friedrich-Alexander-Universität Erlangen-Nürnberg (FAU), 91058, Erlangen, Germany

[4]Department of Physics and Astronomy, Louisiana State University, Baton Rouge, Louisiana 70803, USA

[5]Department of Physics, University of Johannesburg, P.O. Box 524 Auckland Park 2006, Johannesburg, South Africa

[6]Kavli Institute at Cornell for Nanoscale Science, Ithaca, New York 14853, USA

[7]Leibniz-Institut für Kristallzüchtung, Berlin, 12489, Germany

*a.scheid@fkf.mpg.de

Keywords: Delafossites, MBE, interfacial reconstruction, growth mechanism, electron ptychography, heterostructure

Thin films of $PdCoO_2$ were synthesized by shutter-controlled MBE in a Veeco Gen10 MBE system on (001) sapphire and $(111)_{pc}$ $LaAlO_3$ substrates. Details on the film growth can be found in the Materials and Methods section and the Supplementary Material of a previous study[1]. The Al_2O_3 substrates were annealed at 1000 °C for 6 hours prior to growth, resulting in a surface roughness of around 0.08 nm and a step height of about 0.2 nm, as reported in prior work[2].

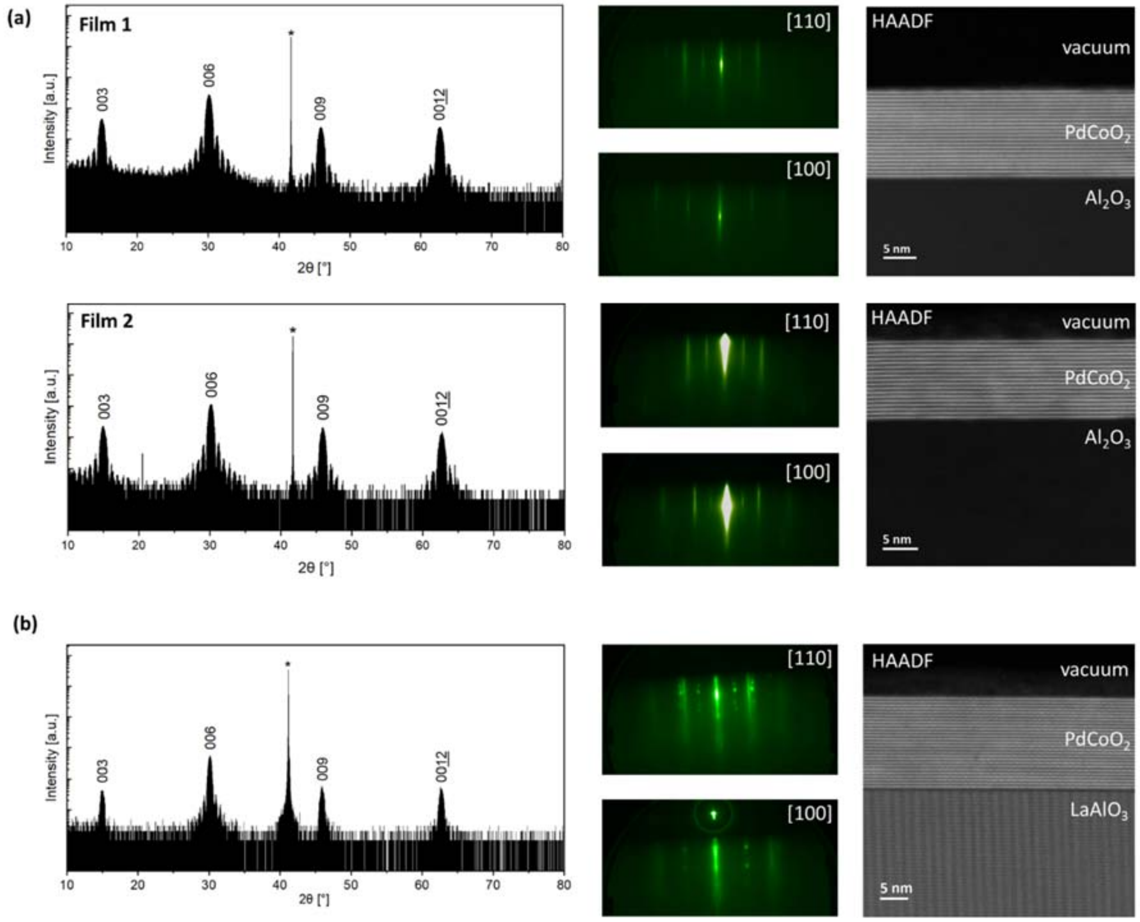

Figure S1. (a) X-ray diffraction, in-situ RHEED patterns, and STEM HAADF images of two $PdCoO_2$ thin films grown on an Al_2O_3 substrate. The Laue oscillations observed for the $PdCoO_2$ peaks indicate a smooth film-substrate interface, which is also evident in the low-magnification STEM images. (b) X-ray diffraction, in-situ RHEED patterns, and STEM HAADF images of a $PdCoO_2$ thin film grown on $LaAlO_3$. Asterisks (∗) denote substrate reflections.

Density Functional Theory (DFT) calculations

DFT calculations were performed using the Quantum ESPRESSO suite[3–5] to assess the likelihood of different $PdCoO_2/Al_2O_3$ interface types. The interface can be most effectively understood by a shared structural feature in the oxygen network: both structures exhibit coinciding oxygen triangles at the interface (yellow triangles in Figure S2(a)), serving as the covalently bonding bridge between the two materials. To construct the DFT unit cells for the interface simulation, rectangular in-plane supercells of both materials were generated, containing an equal number of oxygen triangles. The unit cell vectors $(\vec{a}_S, \vec{b}_S)$ correspond to linear combinations of the primitive cell vectors $(\vec{a}_P, \vec{b}_P)$. For $PdCoO_2$, the chosen supercell was spanned by $\vec{a}_S = 2\vec{a}_P + \vec{b}_P$ & $\vec{b}_S = 3\vec{b}_P$ and for Al_2O_3 by $\vec{a}_S = \vec{a}_P$ & $\vec{b}_S = \vec{a}_P + 2\vec{b}_P$, respectively.

As highlighted in Figure S2(a), the rectangular cells feature two/three oxygen triangles along the short/long side of the rectangle, respectively. To accurately reflect the experimental setup, the in-plane lattice constants of the rectangular simulation cells were fixed to the experimentally determined lattice constant of Al_2O_3. Consequently, the lattice spacing of $PdCoO_2$ (being 2.98 % larger) is slightly compressed compared to its bulk value. For DFT calculations, the construction of periodically continued unit cells is necessary, effectively resulting in a stacked, alternating structure with two identical interfaces per unit cell (see respective unit cell at the bottom of Figure S2(b)). To optimize the structure, we relaxed the lattice constant along the interface normal vector and the atomic positions of all atoms within the unit cell.

The stability of different interfaces was estimated by comparing the total energy of the (optimized) interface and that of its two constituents (see Figure S2(b) - note that the factor two (2Δ) in the equation accounts for the fact that there are two identical interfaces in the simulation cell)[6,7]. The latter was computed in two separate self-consistent field (SCF) calculations from the deconstructed unit cell without any additional geometry optimization. Since the oxygen layer at the interface is shared by both components (Al_2O_3 and $PdCoO_2$), there are, in principle, two ways to define the isolated constituents from the full simulation cell for the calculation of the interface energy (according to the scheme in Figure S2 (b)): the shared oxygen layer can be ascribed to either Al_2O_3 or $PdCoO_2$.

The interface energies were calculated using both ascriptions. The energetic differences between the various interfaces are represented as matrices in Figure S2(c). When the O-layer is ascribed to Al_2O_3 (i.e., the interface energy is defined as the binding energy between the shared O-layer and the Co- or Pd-layer, respectively), we find that the Co-interface is significantly more stable (by 4.0 eV per unit cell area of ~40 $Å^2$). This is reasonable considering that Pd forms covalent bonds with only two oxygen atoms at a 180° bond angle, while Co bonds with six oxygen atoms to form an octahedron (see Figure S2(b)). Consequently, the Co-interface contains three times more Co-O bonds than the Pd-interface has Pd-O bonds, resulting in greater binding energy.

Conversely, in the Pd-interface, each oxygen atom in the shared O-layer is bonded to only one Pd atom, whereas in the Co-interface, each oxygen atom is bonded to three Co atoms. Thus, the shared O-layer in the Pd-interface exhibits a higher demand for electrons from the Al-layer, as the Pd-interface donates less electron density to the shared O-layer compared to the Co-interface. This suggests that the shared O-layer is more strongly bonded to the first Al-layer of Al_2O_3 in the Pd-interface than in the Co-interface, which is confirmed by the binding energies obtained from ascribing the shared O-layer to the delafossite. This can be seen as a direct consequence of the Co-interface being more stable than the Pd-interface in terms of their bonding strength to the shared O-layer.
In addition, different cases of Co-substitution in the first Al-layer of the substrate were tested. The results, presented as 50% Co-substitution (affecting two of the four Al-atoms per Al-layer) and the 100% case, are shown in the antisymmetric matrix in Figure S2(c).

Technical details

For the DFT calculations performed with the Quantum ESPRESSO suite[3–5], optimized norm-conserving pseudopotentials[8,9] were used with an energy cutoff of 120 Ry, and the exchange-correlation interaction was approximated by the Perdew-Burke-Ernzerhof functional (PBE). Brillouin zone integration was carried out on a 6×3×1 electron momentum grid, which corresponds to an equal sampling density for the rectangular supercell with the in-plane lattice constants a=4.7606 Å and b=8.2456 Å. The out-of-plane lattice constant c was larger than 30 Å for all considered simulation cells, such that using only one k-point along the k_z direction is sufficient. A Gaussian smearing of 0.02 Ry was included. The derived total energies were converged within the accuracy of 0.03 eV for the chosen numerical parameters.

During the geometry optimizations, the mentioned in-plane lattice constants, which correspond to the experimental lattice spacing of Al_2O_3, were kept fixed, while the out-of-plane lattice constant (i.e., the stacking direction) and the atomic positions were fully optimized. Different thicknesses of the Al_2O_3 compound in the simulation cells were tested, namely 4, 6, and 8 Al layers. After optimizing these respective simulation cells with different thicknesses of the Al_2O_3 part, the variation in the calculated interface energy was found to be less than 0.01 eV. This confirms that the chosen layer thicknesses (of at least 6 Al layers in the pure, i.e., unsubstituted Al_2O_3) are sufficient to achieve robust and reliable results.

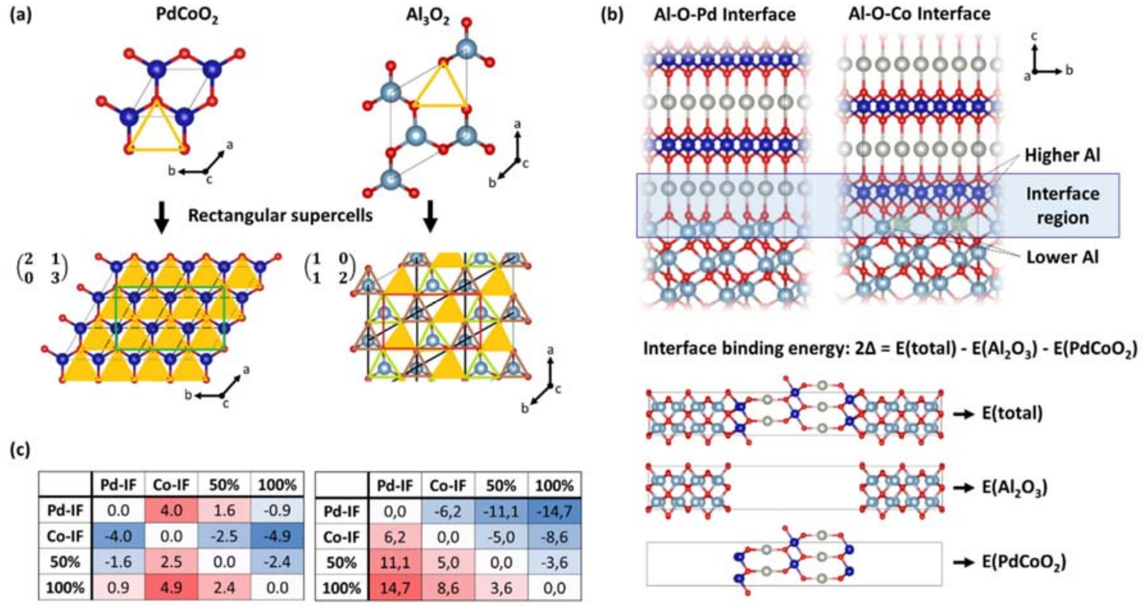

	Pd-IF	Co-IF	50%	100%
Pd-IF	0.0	4.0	1.6	-0.9
Co-IF	-4.0	0.0	-2.5	-4.9
50%	-1.6	2.5	0.0	-2.4
100%	0.9	4.9	2.4	0.0

	Pd-IF	Co-IF	50%	100%
Pd-IF	0,0	-6,2	-11,1	-14,7
Co-IF	6,2	0,0	-5,0	-8,6
50%	11,1	5,0	0,0	-3,6
100%	14,7	8,6	3,6	0,0

Figure S2. (a) Oxygen layers exhibiting a triangular (highlighted) in-plane arrangement, a shared structural feature between $PdCoO_2$ and Al_2O_3. The top panel displays the primitive hexagonal unit cells of the two interfaced compounds, while the bottom panel shows the specific rectangular supercells utilized for the interface simulations. (b) Top panel: Side views of the Pd- and Co-interface simulation geometries. The interface region separating the two compounds is highlighted with transparent blue. Co-substituted Al-sites are highlighted as 'Higher Al' and 'Lower Al' depending on their *c*-axis position. The interface binding energy is calculated as the difference between the total energy of the (optimized) interface and that of its two constituents. (c) Differences in calculated interface energies (per unit cell area of ~40 Å^2) between the (in order) Pd-, Co-, 50% Co-substituted, and 100% Co-substituted interfaces. For 50% Co-substitution, various substitution patterns were tested and averaged in the table for simplicity, as all were energetically positioned between the extreme cases of 0% and 100%. The differences are represented as an antisymmetric matrix, where a negative matrix element (i, j) indicates that the i-interface (row index) is more stable than the j-interface (column index). Matrix elements are color-coded: negative values are shown in blue, positive values in red, with a smooth interpolation between the extreme values. Zero values (on the diagonal) are represented in white. The table on the left corresponds to the calculation of interface energies with the shared oxygen layer attributed to Al_2O_3, and the table on the right to the delafossite ($PdCoO_2$) constituent of the simulation cell, respectively.

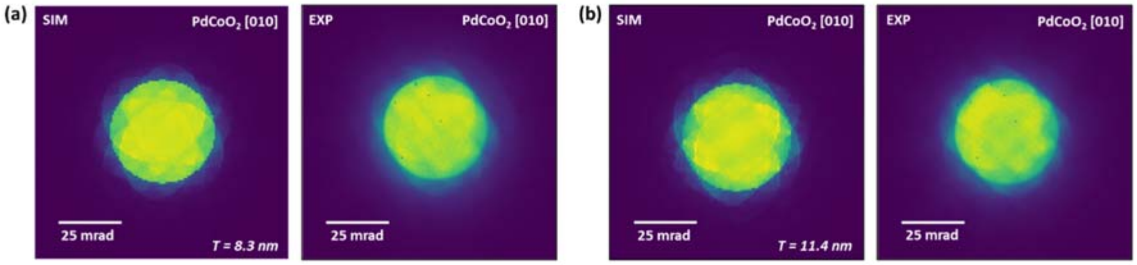

Figure S3. Simulated (SIM) and experimental (EXP) PACBEDs for the [010] zone axis of $PdCoO_2$ from 4D STEM scans across the (a) $PdCoO_2/Al_2O_3$ and (b) $PdCoO_2/LaAlO_3$ interface.

Lattice mismatch calculations

The lattice mismatch quantifies the relationship between the crystalline lattice parameters at the film–substrate interface. In epitaxial thin films, it relates the lattice parameters of the relaxed film and substrate. According to Frank and van der Merwe, the lattice mismatch is given by equation (S1)[10]. For delafossites, growing with a 30°rotation on the substrate (see Figure S4), the in-plane lattice parameter of the film must be corrected by a factor of cos(30°)·2 at the interface.

$$\varepsilon_{\mathrm{m}} = \frac{a_{film} - a_{substrate}}{a_{substrate}} \tag{S1}$$

As a result, for negative values of ε_{m}, i.e. $a_{film} < a_{substrate}$, the films are under tensile strain when growth is initiated and for positive values of ε_{m}, i.e. $a_{film} > a_{substrate}$, the films nucleate under compressive strain on the substrate.

Based on the substrate and film lattice parameters listed below, the following lattice mismatches can be calculated for the systems investigated in this work:

Table S1. Lattice mismatch calculations based on the in-plane lattice parameters of substrate and film.

	$PdCoO_2/Al_2O_3(001)$	$PdCoO_2/LaAlO_3(111)_{pc}$	$PdCoO_2/SrTiO_3(111)_{pc}$
a_{film} [Å]	cos(30°)·2·2.83	cos(30°)·2·2.83	cos(30°)·2·2.83
$a_{substrate}$ [Å]	4.76	5.39	5.51
ε_{m}	+2.98 %	-9.06 %	-11.04 %

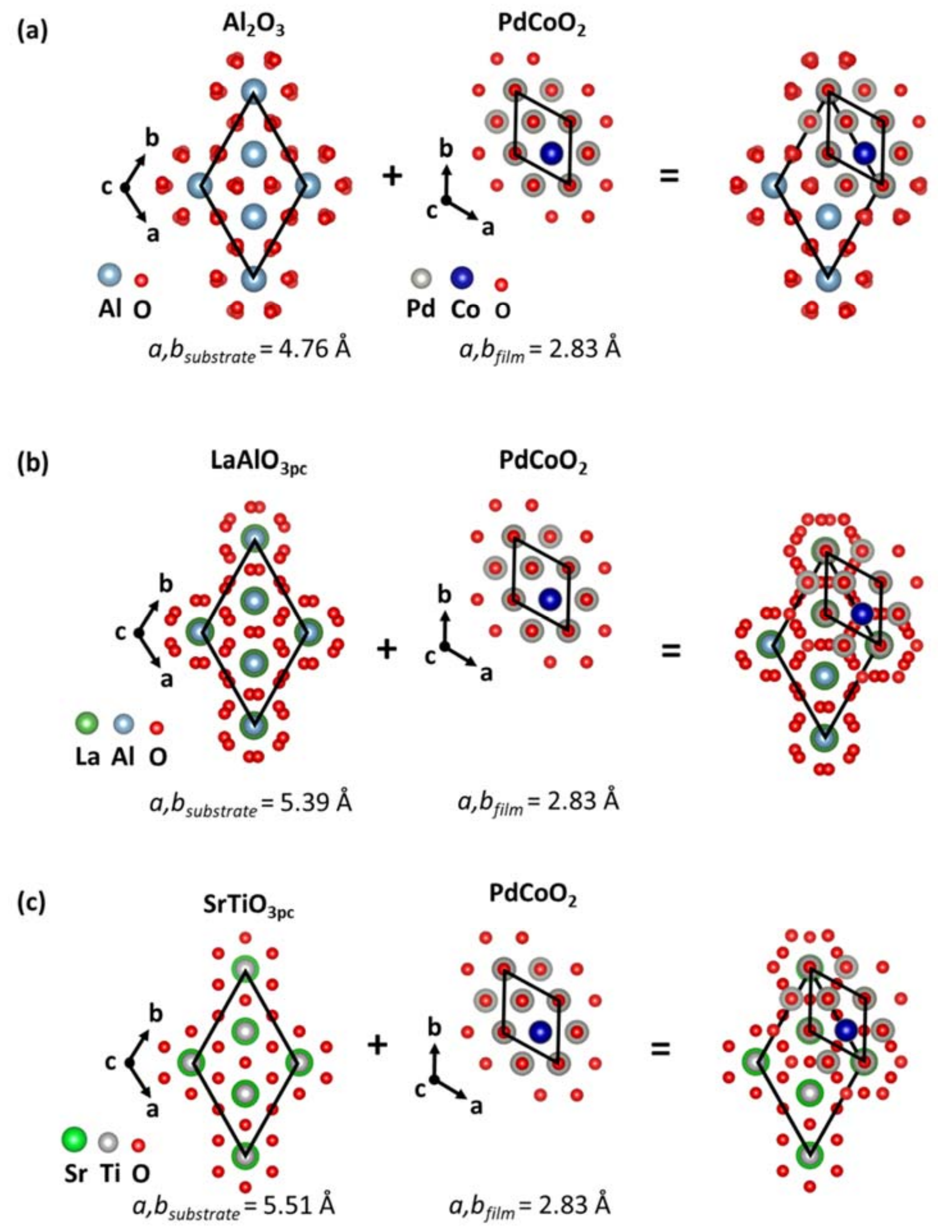

Figure S4. Orientation relationship between the (a) Al_2O_3, (b) $LaAlO_3$ and (c) $SrTiO_3$ substrates and the $PdCoO_2$ thin film with a 30° rotated growth of the film on the substrate. The lattice parameters are given for the solid unit cells indicated in the Figure.

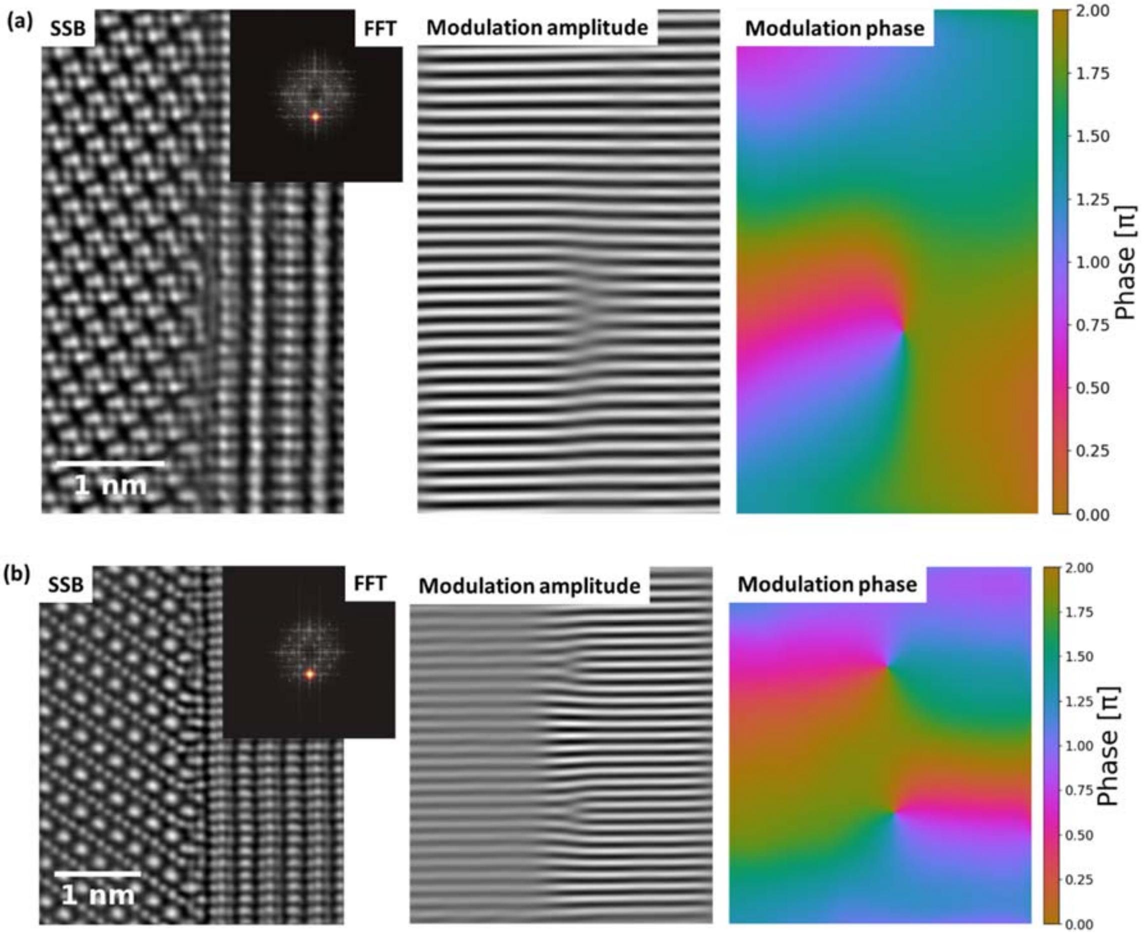

Figure S5. Phase lock-in lattice-modulation analysis from single-sideband phase reconstructions along the [210] zone axis of $PdCoO_2$ on (a) Al_2O_3 and (b) $LaAlO_3$. Extracted modulation amplitude and phase from the fast Fourier transformed image reveal the presence of defects in the interfacial phase.

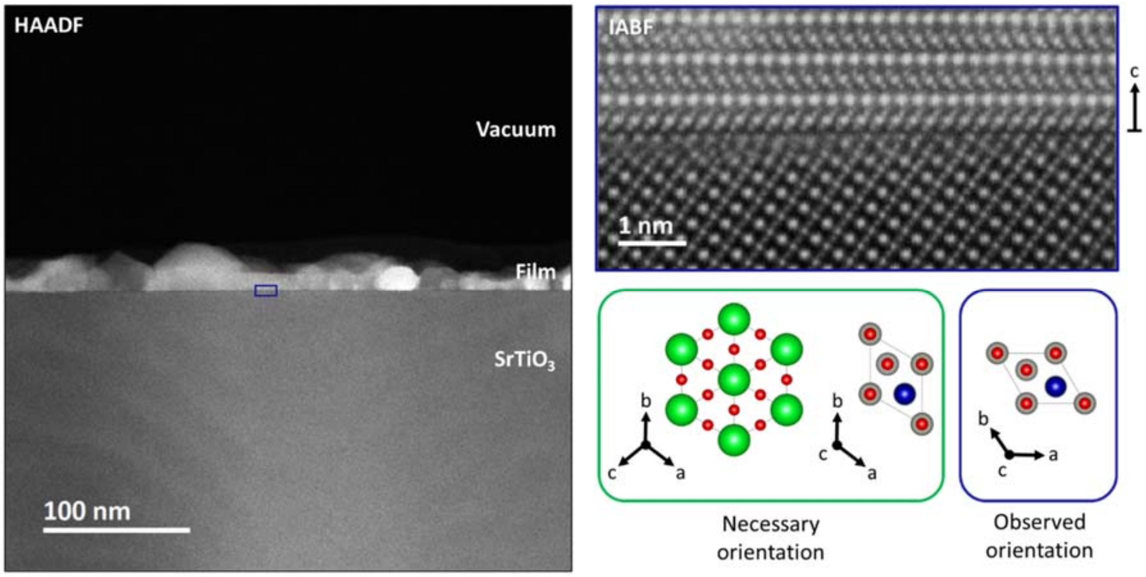

Figure S6. HAADF STEM image of a decomposed $PdCoO_2$ thin film grown on a (111) $SrTiO_3$ substrate. The blue inset highlights an area where decomposition is minimal, revealing a $PdCoO_2$ thin film grown with a 60° rotation instead of the expected 30° rotation on the substrate, as observed in the inverted annular bright-field (IABF) image. The green frame indicates the expected film orientation relative to the (111) $SrTiO_3$ substrate for stable growth, while the blue frame shows the observed orientation along the c-axis of the film.

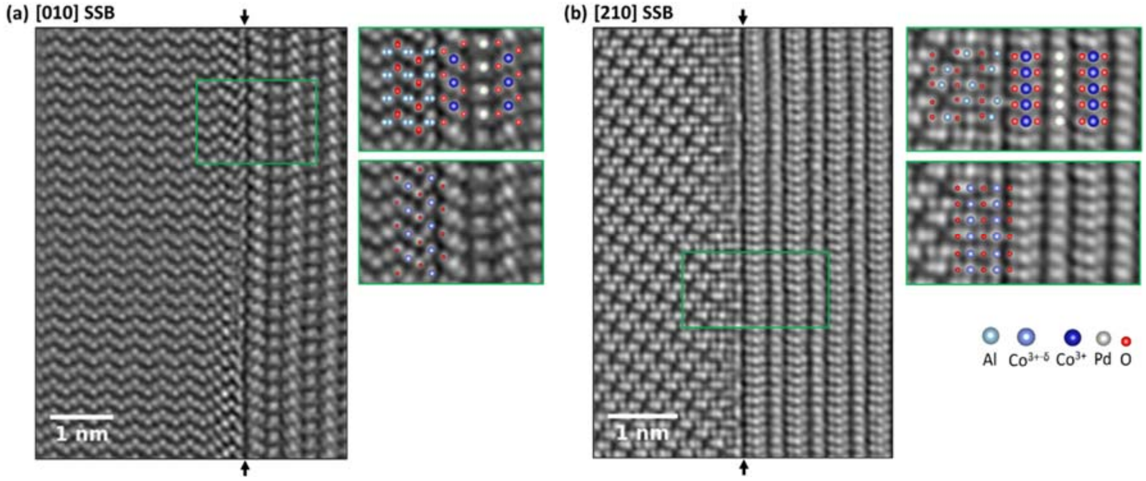

Figure S7. Single-sideband ptychographic phase reconstructions from 4D STEM datasets acquired across the $PdCoO_2/Al_2O_3$ interface for the PLD-grown thin films along the (a) [010] and (b) [210] film orientations. The reconstructions provide strong contrast for all elements and reveal the presence of periodically repeating features within the substrate surface layer. Insets show structural model overlays with the film, substrate, and interfacial phase models along the respective zone axes.

References

1. Song, Q. *et al.* Growth of PdCoO2 films with controlled termination by molecular-beam epitaxy and determination of their electronic structure by angle-resolved photoemission spectroscopy. *APL Materials* **10**; 10.1063/5.0101837 (2022).

2. Sun, J. *et al.* Growth of PdCoO2 by ozone-assisted molecular-beam epitaxy. *APL Materials* **7**; 10.1063/1.5130627 (2019).

3. Giannozzi, P. *et al.* Advanced capabilities for materials modelling with Quantum ESPRESSO. *Journal of physics. Condensed matter : an Institute of Physics journal* **29,** 465901; 10.1088/1361-648X/aa8f79 (2017).

4. Giannozzi, P. *et al.* QUANTUM ESPRESSO: a modular and open-source software project for quantum simulations of materials. *Journal of physics. Condensed matter : an Institute of Physics journal* **21,** 395502; 10.1088/0953-8984/21/39/395502 (2009).

5. Giannozzi, P. *et al.* Quantum ESPRESSO toward the exascale. *The Journal of chemical physics* **152,** 154105; 10.1063/5.0005082 (2020).

6. Welch, E. W., Jung, Y.-K., Walsh, A., Scolfaro, L. & Zakhidov, A. A density functional theory study on the interface stability between CsPbBr3 and CuI. *AIP Advances* **10**; 10.1063/5.0018925 (2020).

7. Jung, Y.-K., Butler, K. T. & Walsh, A. Halide perovskite heteroepitaxy: Bond formation and carrier confinement at the PbS–CsPbBr3 interface. *J. Phys. Chem. C* **121,** 27351–27356; 10.1021/acs.jpcc.7b10000 (2017).

8. Hamann, D. R. Optimized norm-conserving Vanderbilt pseudopotentials. *Phys. Rev. B* **88**; 10.1103/PhysRevB.88.085117 (2013).

9. van Setten, M. J. *et al.* The PseudoDojo: Training and grading a 85 element optimized norm-conserving pseudopotential table. *Computer Physics Communications* **226,** 39–54; 10.1016/j.cpc.2018.01.012 (2018).

10. Frank, F. C. & van der Merve, J. H. One-dimensional dislocations. II. Misfitting monolayers and oriented overgrowth. *Proc. R. Soc. Lond. A* **198,** 216–225; 10.1098/rspa.1949.0096 (1949).
